# Supplementary material for: Vaping and mental health: A cross-sectional study among university students in Bangladesh
Source: PLoS One. 2026 Mar 2;21(3):e0343502. doi: 10.1371/journal.pone.0343502 (PMC12952649; doi:10.1371/journal.pone.0343502)
Supplement: Attachment — Submitted filename: Response to Reviewers_20250706.docx [file pone.0343502.s003.docx]

PONE-D-25-24214

Vaping and mental health: A cross-sectional study among university students in Bangladesh

PLOS ONE

Dear Dr. Sabrina,

Thank you for submitting your manuscript to PLOS ONE. After careful consideration, we feel that it has merit but does not fully meet PLOS ONE’s publication criteria as it currently stands. Therefore, we invite you to submit a revised version of the manuscript that addresses the points raised during the review process.


Please submit your revised manuscript by Aug 08 2025 11:59PM. If you will need more time than this to complete your revisions, please reply to this message or contact the journal office at [plosone@plos.org](mailto:plosone@plos.org). When you're ready to submit your revision, log on to https://www.editorialmanager.com/pone/ and select the 'Submissions Needing Revision' folder to locate your manuscript file.

Please include the following items when submitting your revised manuscript:

- A rebuttal letter that responds to each point raised by the academic editor and reviewer(s). You should upload this letter as a separate file labeled 'Response to Reviewers'.
- A marked-up copy of your manuscript that highlights changes made to the original version. You should upload this as a separate file labeled 'Revised Manuscript with Track Changes'.
- An unmarked version of your revised paper without tracked changes. You should upload this as a separate file labeled 'Manuscript'.

If you would like to make changes to your financial disclosure, please include your updated statement in your cover letter. Guidelines for resubmitting your figure files are available below the reviewer comments at the end of this letter.

If applicable, we recommend that you deposit your laboratory protocols in protocols.io to enhance the reproducibility of your results. Protocols.io assigns your protocol its own identifier (DOI) so that it can be cited independently in the future. For instructions see: <https://journals.plos.org/plosone/s/submission-guidelines#loc-laboratory-protocols>. Additionally, PLOS ONE offers an option for publishing peer-reviewed Lab Protocol articles, which describe protocols hosted on protocols.io. Read more information on sharing protocols at <https://plos.org/protocols?utm_medium=editorial-email&utm_source=authorletters&utm_campaign=protocols>.

We look forward to receiving your revised manuscript.

Kind regards,

Ali Awadallah Saeed
Academic Editor
PLOS ONE

Journal requirements:

When submitting your revision, we need you to address these additional requirements.

1. Please ensure that your manuscript meets PLOS ONE's style requirements, including those for file naming. The PLOS ONE style templates can be found at

https://journals.plos.org/plosone/s/file?id=wjVg/PLOSOne_formatting_sample_main_body.pdf and

<https://journals.plos.org/plosone/s/file?id=ba62/PLOSOne_formatting_sample_title_authors_affiliations.pdf>

**Response:** Thank you for your comments. We addressed this in the revised manuscript. We followed the formatting sample of the author's affiliation and changed it. PLEASE SEE page 1, lines 18 to 19. (Revised Manuscript _20250706_clean file)

2. Your ethics statement should only appear in the Methods section of your manuscript. If your ethics statement is written in any section besides the Methods, please move it to the Methods section and delete it from any other section. Please ensure that your ethics statement is included in your manuscript, as the ethics statement entered into the online submission form will not be published alongside your manuscript.

**Response:** Thank you for your comments. We addressed this in the revised manuscript. All of our ethics statements are only in the METHODS section, PLEASE SEE page 6, lines -173-176. (Revised Manuscript _20250706_clean file)

**Additional Editor Comments:**
Thank you for this important and timely study. I have two points of clarification that I believe would enhance the transparency and rigor of your methodology, before I send the manuscript to reviewers:

University Selection Criteria: Could you please elaborate on the criteria used to select the seven universities across Bangladesh? Specifically, it would be helpful to understand the rationale behind including two private and five public institutions.

**Response:** Thank you for your comments. We addressed this in the revised manuscript. we selected these seven universities across Bangladesh. We employed convenience sampling based on the known networks of the investigators to recruit seven universities. PLEASE SEE page no-4, line no-108, 109. (Revised Manuscript _20250706_clean file)

Use of Standardized Assessment Tools: Kindly clarify whether permission or ethical approval was obtained for the use of the standardized tools in your study, namely the Kessler Psychological Distress Scale (K-10), the CESD-10, and the GAD-7. Indicating this would strengthen the ethical integrity of the research.

**Response:** Thank you for your comments. We addressed this in the revised manuscript. These three scales, namely, the Kessler Psychological Distress Scale (K-10), the Center for Epidemiologic Studies Depression Scale (short form) (CESD-10), and the Generalized Anxiety Disorder scale (GAD-7), are freely available in public domains and do not require any permission for usage for scientific purposes. As such, no additional permission or alike was required for the usage of these scales in this study. Please refer to the following sources for confirmation.

1.https://www.hcp.med.harvard.edu/ncs/k6_scales.php

2.https://eprovide.mapi-trust.org/instruments/center-for-epidemiologic-studies-depression-scale-10

3.https://www.pfizer.com/news/press-release/press-release-detail/pfizer_to_offer_free_public_access_to_mental_health_assessment_tools_to_improve_diagnosis_and_patient_care

Furthermore, we obtained ethics approval from the Institutional Review Board (IRB) of North South University in Bangladesh, with the reference number 2020/OR-NSU/IRB/1104. This approval would have permitted the use of these standardized tools in this study. PLEASE SEE, page no -5,6. line no -148 to 153. (Revised Manuscript _20250706_clean file)

[Note: HTML markup is below. Please do not edit.]

Reviewers' comments:


[NOTE: If reviewer comments were submitted as an attachment file, they will be attached to this email and accessible via the submission site. Please log into your account, locate the manuscript record, and check for the action link "View Attachments". If this link does not appear, there are no attachment files.]

While revising your submission, please upload your figure files to the Preflight Analysis and Conversion Engine (PACE) digital diagnostic tool, <https://pacev2.apexcovantage.com/>. PACE helps ensure that figures meet PLOS requirements. To use PACE, you must first register as a user. Registration is free. Then, login and navigate to the UPLOAD tab, where you will find detailed instructions on how to use the tool. If you encounter any issues or have any questions when using PACE, please email PLOS at [figures@plos.org](mailto:figures@plos.org). Please note that Supporting Information files do not need this step.
