# Supplementary material for: Vaping and mental health: A cross-sectional study among university students in Bangladesh
Source: PLoS One. 2026 Mar 2;21(3):e0343502. doi: 10.1371/journal.pone.0343502 (PMC12952649; doi:10.1371/journal.pone.0343502)
Supplement: Attachment — Submitted filename: Response to reviewers_comments_20251221_.docx [file pone.0343502.s004.docx]

**Response to reviewers**

**Vaping and mental health: A cross-sectional study among university students in Bangladesh**

*The line numbers and page numbers are mentioned according to the track-changed version.*

| **Comments from Reviewer-1** | **Responses** |
| --- | --- |
| **Introduction:** |  |
| The introduction contains several grammatical errors, awkward phrasings, and wordy constructions that make reading difficult. There are issues with sentence structure and clarity in multiple instances. | Thanks for your comments. The introduction has been thoroughly revised for grammatical accuracy, sentence flow, and conciseness. Redundant or awkwardly phrased sentences have been removed to improve readability and coherence. |
| There is a significant inconsistency in reported vaping prevalence in Bangladesh (from 0.2% to 31.27%) without sufficient explanation of methodological differences between studies. This weakens credibility and may confuse readers. | We agree that the large variation in reported vaping prevalence requires further clarification. In the revised manuscript, we have added an explanation highlighting the methodological and contextual differences between studies. Specifically, “ the Global Adult Tobacco Survey (GATS) 2017 reported a low overall vaping prevalence of 0.2% among both males and females [9]. In contrast, a study conducted during the COVID-19 pandemic reported a much higher prevalence of 31.3% among young adults [10]. This discrepancy likely arises due to two data collection methods. The methodological differences between the two studies were (GATS-A multistage, clustered sampling design for nationally representative data). and the COVID-19 pandemic article was done by (a convenient sampling technique and online self-reported survey, the timing of data collection during the COVID-19 pandemic, as well as the increasing social acceptability and awareness of vaping in recent years). These explanations have been incorporated into the introduction (page 3, line 88-91, Page 4, line 92-94) to improve clarity and strengthen the credibility of the findings. |
| Several ideas are repeated unnecessarily, such as the harmful effects of nicotine and health risks for pregnant women. These could be condensed to maintain reader engagement and clarity. | Thanks for your comments. In the revised manuscript, we have removed the repeated ideas. (Please see lines 106-108, page no 4). |
| The paragraph progression lacks smooth transitions between global trends, regional context, and local findings. The narrative jumps between prevalence, physical effects, and mental health without clear thematic segmentation. | Thank you for this valuable comment. We have revised the introduction to improve the logical flow and ensure smoother transitions between global, regional, and local contexts. The revised version now follows a clear progression: It begins with global trends in vaping prevalence (USA, Canada, Australia, and other high-income countries), then moves to regional patterns in Asia and South Asia, and finally focuses on the Bangladeshi context. To maintain clarity, we have also reorganized the discussion of health impacts into two separate segments, first outlining physical health consequences (e.g., nicotine addiction, respiratory and cardiovascular risks), and then introducing the mental health dimension (e.g., stress, anxiety, depression, and substance co-use). Please see lines 66-114.page-3,4 |
| There is no theoretical basis or explanatory model provided to support the hypothesized relationship between vaping and mental health. This weakens the foundation for the research objectives. | Thank you for this valuable comment. In the revised manuscript, we have added a theoretical explanation for the relationship between vaping and mental health, which can be grounded in biological and psychological mechanisms. (Please check lines 115-132, pages 4,5). |
| While the introduction mentions a lack of studies from developing countries, it does not clearly articulate what specific aspect is missing in current literature or how this study uniquely addresses it. | We agree that the research gap needs to be more clearly specified. In the revised introduction, we have clarified that although previous studies, mainly from high-income countries, have focused on the prevalence, reasons for use, and physical health impacts of vaping, there is a notable lack of evidence exploring its mental health correlates, particularly in low and middle-income countries (LMICs) such as Bangladesh. To address this gap, we have emphasized that the present study is among the first to examine the association between vaping and mental health outcomes, specifically psychological distress, anxiety, and depression, among university students in Bangladesh. This focus provides new insights into an underexplored population and contributes to the global understanding of vaping-related mental health risks in developing contexts. (please see line156-167, page 6) |
| Some claims, such as describing post-COVID vaping as an “epidemic,” are overstated or emotive without sufficient supporting evidence, affecting the academic tone of the introduction. | Thank you for bringing this to our attention. We agree that the use of the term “epidemic” was overly strong and could be misleading without sufficient evidence. To address this, we have revised the relevant section of the introduction to adopt a more objective and evidence-based tone. The sentence now reads: “More recent data, however, suggest a sharp rise. A study conducted during the COVID-19 pandemic found a vaping prevalence of 31.3% among young adults [10]. The considerable discrepancy between these findings may reflect differences in target populations, data collection methods, and the evolving social acceptability of vaping rather than an actual epidemic-level surge.” (Please see page 3,4, line 91-94) |
| The objective of the study is presented at the end but could be more concisely stated and clearly linked to the identified research gap. | Thank you for this helpful comment. We have revised the final paragraph of the introduction to state the study objective more concisely and to ensure a clear connection with the identified research gap. The revised version now directly links the limited evidence on vaping and mental health outcomes among young adults in Bangladesh to the purpose of the current study.  Specifically, the revised objective now reads: “This study aimed to examine the association between vaping and mental health outcomes, specifically psychological distress, anxiety, and depression, among undergraduate university students in Bangladesh. This study explores how behavioral factors (vaping), psychological factors (mental health symptoms), and social (youth lifestyle and peer influence) factors interact within this emerging public health issue. please check (lines161-167, page-6) |
| **Methods** |  |
| Lack of information about how participants were distributed across universities and demographic strata raises concerns about selection bias. | We appreciate your review. We have clarified the distribution of participants across the seven universities in the revised method section. Among the seven universities, data were conveniently collected based on geographic representation and accessibility through the investigators’ collaboration networks. Geographically, students were recruited from four major cities in Bangladesh named Dhaka, Chittagong, Sylhet, and Khulna. (please see page 6, lines 171-179). We also acknowledged potential selection bias in the limitations of the discussion section. |
| No mention of any blinding, data validation, or procedures to reduce response bias in self-administered surveys. | Thank you for reviewing. We have added additional information under the section Data Collection (section 2.4). We conducted a pilot study with a small number of university students once the questionnaire was prepared to check the language of the survey. We did necessary modifications in languages before finalising the final version of the questionnaire. The survey was anonymous and self-administered without adding any personal data to minimize response bias. Participants were assured of their confidentiality. The Google Form was configured to allow only one submission per email to avoid double submission. The Google Form required completion of all mandatory fields, and participants could not proceed to the next section without completing each part. Therefore, there were no missing responses, minimizing the possibility of missing data. Before analysis, all responses are screened for completeness and duplicate entries”. As this was an anonymous online survey, blinding was not applicable. Please see lines 203-212, page 7 |
| The assumed 30% vaping prevalence is based on anecdotal evidence, which weakens the scientific rigor of the sample size justification. | We appreciate your review. The sample size was calculated using OpenEpi. Here, we addressed your review by adding survey findings and reports from South Asian countries data Please see the reference no 8. Considering that prevalence along with 95% confidence intervals (CIs), and 80% power, the estimated minimum sample size was 323. Convenient sampling was used to recruit the study participants. Please see lines 186-192, page 7 |
| Repetition occurs across several parts of the section (e.g., ethical approval is mentioned twice, phrasing redundancies in informed consent). | Apologies for oversight. We have revised the manuscript as per your comments and removed repetitive mentions of ethical approval. |
| Descriptions of K-10, CESD-10, and GAD-7 are overly long and not consistently formatted. | Thank you for your valuable feedback. In the revised manuscript, the descriptions of the Kessler Psychological Distress Scale (K-10), the Center for Epidemiologic Studies Depression Scale (CESD-10), and the Generalized Anxiety Disorder Scale (GAD-7) have them more concise and consistently formatted. Please check lines 220-231, page 8. |
| There's a factual inconsistency in referring to the CESD-10 but citing a source intended for the CESD-20 or CESD-R. | We appreciate your observation and have now added another reference for CSD-10.ref no-26 *Andresen EM, Malmgren JA, Carter WB, Patrick DL. Screening for depression in well older adults: Evaluation of a short form of the CES-D. American Journal of Preventive Medicine. 1994;10(2):77–84*. Please check line 225, page -8 |
| The rationale behind the logistic regression modeling approach and the threshold (p < 0.20) for variable inclusion lacks clarity and justification. | Thank you for your comment. In the revised manuscript, we added references for clarity and justification of the rationale behind the logistic regression modelling approach and the threshold (p < 0.20). we have added ref no 28,29. Please check the references.  *28.Rahman MA, Wilson AM, Sanders R, Castle D, Daws K, Thompson DR, et al. Smoking behavior among patients and staff: a snapshot from a major metropolitan hospital in Melbourne, Australia. Int J Gen Med.2014;7:79-87. doi:10.2147/IJGM.S54230.*  Please see lines 265-267, page 9 |
| No detail is provided on handling missing data or response validation for online survey submissions. | We have clarified in the Data Collection (section 2.4). The survey was anonymous and self-administered without adding any personal data to minimize response bias. Participants were assured of their confidentiality. The Google Form was configured to allow only one submission per email to avoid double submission. The Google Form required completion of all mandatory fields, and participants could not proceed to the next section without completing each part. Therefore, there were no missing responses, minimizing the possibility of missing data. Before analysis, all responses are screened for completeness and duplicate entries. Please see lines 206-212, page 7 |
| Ethical approval and consent information are repeated unnecessarily. | In the revised manuscript, we removed this. |
| Specific details about participant protections (e.g., data encryption or access controls) are not described. | In the revised manuscript, we add this. Please see section 2.7 Ethics, lines 275-280, page 10 |
| There are formatting inconsistencies. | In the revised manuscript, we corrected the formatting issues. |
| **Results** |  |
| Several statements repeat information already shown in tables (e.g., prevalence rates of psychological distress, depression, and anxiety among vape and dual users). Repetition of percentages and exact numbers without analytical interpretation weakens narrative flow. | We have revised the Results section to remove repetitive statements. Percentages and numbers are now presented only once and aligned with the corresponding tables. Please check the description of Table 1 and Table 2 |
| Terms such as “vaping only-1” and “vaping only-2” are unclear and inconsistently labeled across tables and text. | We have revised the terminology for clarity and consistency throughout the manuscript. Specifically, “vaping only-1” has been replaced with **“current vape users with no prior smoking history”**, and “vaping only-2” has been replaced with **“current vape users with previous smoking history.”** These updated terms are now used consistently in all tables and text. Please check (Table 2) |
| Typographical issues such as misplaced line breaks and disjointed headings (e.g., Table 2 and vaping descriptions) interrupt readability. | We have carefully reviewed and corrected the typographical and formatting issues throughout the manuscript. Table 2 and related sections have been reformatted for improved readability and consistency, with clear headings and proper alignment. Please check Table 2 |
| Data analysis leans mostly on descriptive statistics without a clear transition to inferential results. | In the revised manuscript, we divided the result section into two subgroup analysis for your convenience. the e result section provides a clearer transition from descriptive to inferential analyses.  Please check page no 10, line 286, and page 15, line 310. |
| The rationale for selecting variables into multivariate models is not consistently explained | In revised manuscript, We have added a clear description of variable selection criteria in the Methods and Results sections. we added a new reference for your convenience.  Rahman MA, Wilson AM, Sanders R, Castle D, Daws K, Thompson DR, et al. Smoking behavior among patients and staff: a snapshot from a major metropolitan hospital in Melbourne, Australia. Int J Gen Med. 2014;7:79-87. doi:10.2147/IJGM.S54230.Stepwise logistic regression was applied, and variables with p < 0.20 in univariate analyses were included in multivariate models. Covariates were selected based on theoretical relevance and prior literature. please check the references 28, page 9, line- 265-267 |
| Tables show adjusted and unadjusted odds ratios, but variable inclusion criteria in multivariate models are only briefly referenced at the bottom of each table and inconsistently reported | We have now clearly and consistently described the variable inclusion criteria for all multivariate models in both the *Methods* section and the table footnotes in the review manuscript. Specifically, variables with *p* < 0.20 in univariate analyses, along with theoretically important confounders (gender, academic year, university type, socioeconomic status, alcohol consumption, recreational drug use, diagnosed mental health problem, and relevant psychological scales), were entered into the multivariate logistic regression models. Please check page 19 (lines 327-330), page -22,23, (lines 341- 346), page 26 (lines 358-361) |
| Some CIs and p-values do not align well with conclusions drawn (e.g., borderline significance interpreted as definitive associations). | We have re-examined all odds ratios, confidence intervals, and *p*-values in Tables 3–6 and revised the text to ensure interpretations align with the statistical evidence. In the revised manuscript, we removed all the Borderline significance associations. (Please see Table 4, university type removed in the revised manuscript) |
| There is a lack of clarity on whether mental health outcomes are predictors or outcomes of vaping, especially in subgroup analyses. | We have clarified in the Methods, Results, and Discussion sections that mental health variables (psychological distress, depression, and anxiety) were analyzed as *predictor variables* rather than *outcomes* of vaping or dual use. Given the cross-sectional nature of our data, we cannot conclude causality. Thus, the observed associations indicate correlations only. We have added a statement in the Discussion section, acknowledging that the directionality between vaping and mental health outcomes cannot be determined. Longitudinal studies are needed to explore the potential causal relationship. line 506-515 page 31, |
| The role of diagnosed mental health conditions is inconsistently interpreted—sometimes associated with decreased vaping, other times not explained. | The interpretation of the “diagnosed mental health problem” variable has been clarified for consistency in the revised manuscript. Please check lines 318-319, page 15. |
| Subgroup analysis for individuals without pre-existing mental health conditions (Table 6) lacks a clear rationale and may inflate Type I error without proper correction or framing. Transitions between narrative results and table interpretations are abrupt and sometimes fragmented. | Thank you for this thoughtful comment. Our study specifically aimed to examine the mental health correlates of vaping, focusing on psychological distress, anxiety, and depression among university students. Previous research shows that pre-existing mental health conditions can confound these associations; we conducted a subgroup analysis restricted to students without prior diagnoses to assess whether the relationships observed in the full sample persisted independent of baseline psychological vulnerability. We have now clarified this rationale in the manuscript. We agree that additional subgroup analyses may increase the potential for Type I error. To address this, we have explicitly acknowledged in the revised text that this analysis is exploratory and that the findings should be interpreted with caution.(Page 9, line 262-264) |
| **Discussion** Key ideas, such as the prevalence of vaping in Bangladesh and its rise during COVID-19 are repeated with slight rewording | Repeated statements regarding vaping prevalence in Bangladesh and during COVID-19 have been consolidated into a single, concise paragraph. Redundant wording has been removed, and long paragraphs with multiple ideas have been split for clarity. (please see page 27, line 371-380) |
| The association between vaping and mental health is revisited multiple times across different paragraphs without offering new insights. | Thank you for your comment. In the revised manuscript, we have consolidated all discussion of the association between vaping and mental health into a single, focused section. Redundant mentions have been removed, and the section now emphasizes interpretation of our findings, consideration of confounding factors, rather than repeating descriptive results. This improves clarity and ensures that each paragraph provides new insights. Please see lines 506-515 on page 31 |
| Citations are presented back-to-back with little synthesis or interpretation. | In the revised manuscript, we have tried to give an interpretation all over in the discussion section |
| External studies are described at length, but their connection to the study’s specific findings is not always clearly established. | Thank you for your comment. In the revised manuscript, External studies are now integrated and synthesized with our findings rather than listed sequentially. Comparisons with other countries, gender and socioeconomic differences, and dual use are clearly linked to the study results. Please check the discussion section. Please see 473-475, page 30 |
| Some statements imply causal links (e.g., “compulsion to follow trends” or “mental health could increase vaping”) despite the cross-sectional design. | Thank you for your valuable observation. We acknowledge that the cross-sectional design of our study does not allow for causal inferences. We have carefully revised the manuscript to avoid any wording that implies causality. Please see lines 396-398, page 28, and we also removed “mental health could increase vaping” the sentences. Please see lines 511-515, page 31 |
| Phrases like “which is a big public health concern” are emotionally charged and could be more neutrally framed. | Thank you for your comment. In the revised manuscript. Emotionally charged phrases, such as “which is a big public health concern,” were replaced with neutral, evidence-based wording. Causal language has been removed, and all statements now reflect associations, consistent with the cross-sectional study design. Please see lines 467-468, page 30. |
| Findings from univariate analysis are emphasized even when multivariate analysis showed no significance. Contradictions occur when interpreting mental health variables as significant in some places but not in others without resolving the inconsistency. | Thank you for this comment. In the revised manuscript, we have addressed it and added significant results of the multivariable logistic regression model, and we removed all the univariate logistic regression model results to solve the inconsistency. |
| Long paragraphs with multiple ideas (e.g., discussing sex, socioeconomic status, and regional comparisons) affect flow and readability. | Thank you for this comment. In the revised manuscript, we have reorganized the Discussion section into shorter, thematically focused paragraphs. Each paragraph now addresses a single topic—such as sex differences, socioeconomic factors, or regional comparisons—to improve clarity, readability, and logical flow. Please see (lines 427-434, page 29), (Lines 422-426, page-28,29) |
| Subtopics such as “dual use,” “alcohol and drug use,” and “socioeconomic status” are not clearly delineated. | Thank the reviewer for this observation. In the revised manuscript, we have reorganized the Discussion section under clearly defined subheadings, including *Dual Use of Cigarettes and Vaping*, *Alcohol and Recreational Drug Use*, and *Sociodemographic Correlates*. This ensures that each subtopic is clearly delineated, improving structure, readability, and comprehension. Please see lines 464 and 490, pages 30,31. |
| Terms like “non-users” are inconsistently defined, sometimes meaning non-vapers and other times meaning non-smokers/vapers. | Thank you for pointing this out. We agree that the term “non-users” was used inconsistently in the earlier version of the manuscript. We have now clearly defined “non-users” to refer specifically to “*non-vapers”* and have ensured consistent usage of this term throughout the revised manuscript. |
| Occasional grammar and syntax issues (e.g., “Contiguity of smoking environments” or “the odds of drinking alcohol were 11 times higher for non-users”). | Thank you for your comments. In the revised manuscript, we addressed it. we removed it. Please check line 385. page 27, and we added, Alcohol and recreational drug use were high among the participants who reported vaping and dual smoking in this study. The odds of drinking alcohol were 11 times higher among non-vapers compared to vapers. please check the lines 491-492, page 31 |
| Explanations for non-significant results in multivariable analyses are brief and speculative. | we remove all the non-significant results in revised manuscripts. only significant multivariable logistic regression results are added. |
| The role of confounding variables is mentioned but not explored in depth (e.g., impact of alcohol/drug use on mental health and vaping). | Thank you for this observation on confounding factors, including alcohol and recreational drug use, which are now discussed in depth, explaining why associations between vaping and mental health outcomes were not significant in multivariate analyses. please see lines 498-505, page no-31 |
| Neurobiological explanations for the nicotine mental health link are mentioned but lack detail or citation depth. | Thank the reviewer for this observation. Neurobiological explanations for nicotine’s impact on neurotransmitter systems and mental health outcomes have been expanded and appropriately cited to provide a stronger conceptual foundation, please see lines 476--489, page 30 |
| **Limitations** Statements like “this warrants strict public health intervention” are not linked to specific policy or intervention models. | Thank you for your thoughtful comment. We agree that our earlier statement was too general. In the revised manuscript, we added these things in the conclusion section. we have specified potential policy and intervention approaches relevant to the Bangladeshi university context. Specifically, we now suggest implementing mobile health (mHealth)–based smoking cessation and counselling programs, integrating vaping prevention into existing tobacco control initiatives, and strengthening on-campus mental health and peer-support services. Please see lines 547-554, page 32. |
| Biases like recall bias, selection bias, and the impact of social desirability are acknowledged but not critically examined. | Thank you for this insightful comment. In the revised manuscript, we have expanded the Limitations section to more thoroughly address how recall bias, reporting bias, selection bias, and social desirability bias have influenced our findings. We also note that recall bias have affected participants’ ability to accurately report their past behaviors, and that using a convenience sample of university students may limit the generalizability of our results. These revisions provide a more critical assessment of potential biases and their implications for interpreting the study’s findings.  please see lines 517-535, pg-31,32 |
| No mention of potential underrepresentation of female users due to cultural factors. | The female underrepresentation of female users in any observational studies in the Bangladesh context is probably due to cultural or religious, or social factors. We have now added references. Please see 424-426, page 28, 29  ref no 37: *Alahmadi, S. M., & Al-Zalabani, A. H. (2025). Patterns and Factors Associated with E-Cigarette Initiation and Transition Among University Students in Al-Madinah City, Saudi Arabia: A Cross-SectionalStudy. Healthcare, 13(16),1949. https://doi.org/10.3390/healthcare13161949* |
| **Conclusion** The conclusion largely reiterates prior points without introducing a distinct final perspective. | Thank you for your feedback. In the revised manuscript, the Conclusion has been restructured to provide a distinct final perspective, emphasizing the novel contribution of this study. Please see lines 541-554, page 32 |
| Recommendations for university mental health policies are broad and not grounded in study findings or feasible actions. | Thank you for your thoughtful comment. We agree that our earlier statement was too general. In the revised manuscript, we have specified potential policy and intervention approaches relevant to the Bangladeshi university context. Specifically, we now suggest implementing mobile health (mHealth)–based smoking cessation and counselling programs, integrating vaping prevention into existing tobacco control initiatives, and strengthening on-campus mental health and peer-support services. These revisions clarify how the study findings can inform actionable public health and university-level interventions. Please see lines 547-554, page 32. |
| **Comments from Reviewer 2** | **Responses** |
| -First, there’s several studies that examine the use of vape and mental health even in developing countries. I advise the authors to focus their aim in literature (novel aspect) on Bangladesh settings, if there’s another novel aspect please correct me and consider it. | Thank you for your comment. In response, we have revised the Introduction to highlight that, while research on vaping and mental health exists in other contexts, there is a lack of evidence from Bangladesh, where vaping is a relatively new and emerging behavior among youth. Our study is among the first to explore the association between vaping and mental health status among university students in Bangladesh. This provides context-specific insights into behavioral and psychosocial determinants within a South Asian setting, contributing to the limited literature from low- and middle-income countries. These revisions clarify the unique contribution and contextual relevance of our study.  please see line 156-167, page -6. |
| - Can you justify the selection of these 7 universities? | We appreciate your review. We have clarified about participants' distribution across the seven universities in the revised method section. Among the seven universities, two were private and five were public, and were conveniently selected based on geographic representation and accessibility through the investigators’ collaboration network. according to the known networks of the study investigators. Both private universities and two public universities were located in the capital city of Dhaka, and the other three public universities were selected from the three large metropolitan areas of Chittagong, Sylhet, and Khulna in Bangladesh. Please check lines 172-179, page 6 |
| - In the study population you mentioned “who could respond to an English questionnaire” can you justify this? How is this not biased to the study population? As I know, the English language is not the official Bangladesh language. | We appreciate your review. In Bangladesh, English is the primary medium of instruction at the university level, both public and private. Therefore, students enrolled in tertiary education possess adequate proficiency in English to comprehend and accurately respond to survey items. Additionally, the questionnaire underwent pretesting to ensure clarity and contextual relevance, and no participants reported difficulty understanding the items. Given these considerations, we believe that the use of an English questionnaire did not introduce substantial selection or response bias within this study population. Please see page 7, lines 203-205 |
| - Can you add more context about the Open Epi and cite it. | The sample size for this study was calculated using OpenEpi (version 3.01), an open-source web-based epidemiologic and statistical tool designed for epidemiologic calculations, including sample size estimation, confidence intervals, and hypothesis testing. OpenEpi is widely used in public health research due to its transparency, accessibility, and reliability. The sample size was calculated based on an estimated prevalence of e-cigarette use among university students from prior studies, with a 95% confidence level and 5% margin of error. In our revised manuscript, we added a reference: 23. *Dean AG, Sullivan KM, Soe MM. OpenEpi: Open Source Epidemiologic Statistics for Public Health, Version 3.01. Available from:* [*https://www.openepi.com*](https://www.openepi.com)*. please check 186, page 7* |
| - Your sample size was 323 and your final sample was about 1,600 in a data collection period of 10 months. Can you explain this? What was your aim longitudinal study? | While the initially calculated minimum sample size was 323, the final dataset included 1,615 respondents collected over 10 months. This increase resulted from the use of an online survey distributed across multiple universities to enhance representativeness and statistical power. The larger sample allowed for more robust subgroup analyses and improved precision of estimates. The study was cross-sectional, not longitudinal. The extended data collection period was due to logistical factors, such as staggered academic schedules across universities and the time required to obtain administrative permissions and participant responses. Each participant completed the survey only once, and no follow-up data were collected. Hence, the study design remained cross-sectional, despite the prolonged data collection timeframe. |
