# Supplementary material for: Vaping and mental health: A cross-sectional study among university students in Bangladesh
Source: PLoS One. 2026 Mar 2;21(3):e0343502. doi: 10.1371/journal.pone.0343502 (PMC12952649; doi:10.1371/journal.pone.0343502)
Supplement: Attachment — Submitted filename: Response to Reviewers.docx [file pone.0343502.s005.docx]

**Response to Editor Comments:**

Thank you for the insightful remarks. We appreciate the advice and comments, which undoubtedly improve the quality of our manuscript.

**Editor Comment 1**

**Comment:**

If the reviewer comments include a recommendation to cite specific previously published works, please review and evaluate these publications to determine whether they are relevant and should be cited. There is no requirement to cite these works unless the editor has indicated otherwise.

**Response:**
Thank you for your comment. We carefully reviewed all reviewer-recommended references. Relevant and scientifically appropriate publications have been incorporated into the manuscript. References that were not directly aligned with the study objectives or context were not included.

**Editor Comment 2**

**Comment:** Please review your reference list to ensure that it is complete and correct. If you have cited papers that have been retracted, please include the rationale for doing so in the manuscript text, or remove these references and replace them with relevant current references. Any changes to the reference list should be mentioned in the rebuttal letter that accompanies your revised manuscript. If you need to cite a retracted article, indicate the article’s retracted status in the References list and also include a citation and full reference for the retraction notice.
**Response:**
We carefully reviewed all the references for completeness, accuracy, compliance with journal guidelines, and retraction status using publisher websites, Cross Ref, and PubMed records.

- No retracted articles were identified among the cited references; therefore, no retraction notices were required to be added, and no references were removed for this reason.
- Several technical corrections were made to improve the accuracy and integrity of the reference list, including the removal of duplicate entries. Specifically, References 34, 39, and 41 were identified as duplicates and have therefore been removed from the track-changed version. The references in the Discussion section have been reorganized to maintain correct order and consistency with the updated reference list, both the revised manuscript and the track-changed version.
- There are some corrections of DOIs and formatting errors, which have also been solved. In the track changed version, in References 2 and 38, their respective DOIs have been formatted. In the clean version and track change version, we also addressed it accordingly.
- Harmonization was done between journal titles and citation style to comply with PLOS ONE guidelines.

These changes have been implemented in the revised manuscript and are visible in the tracked-changes file.

**Additional Editor Comments**

**Comment:**
The sample-size calculation assumes a 30% prevalence of vaping among university students in Bangladesh, described as anecdotal in the absence of available data. Could you clarify the scientific basis for selecting this value? Please indicate whether it was derived from published studies, pilot data, or a conservative estimation approach, and consider providing an appropriate reference or justification.

**Response:** Thank you for this important methodological question. We have clarified and strengthened the justification for the assumed 30% prevalence used in the sample size calculation. At the time of study design, no nationally representative data were available on vaping prevalence among Bangladeshi university students. Therefore, we adopted a conservative prevalence estimate informed by published evidence from comparable South Asian populations. We considered regional evidence from South Asia, where vaping prevalence among young adults and university students has been reported to be high. For example, a study from India reported a vaping prevalence of 23% among young people (please see 187-188 lines and page no. 7 on the track change version). We have now revised the Methods (Section 2.3: Sampling) to explicitly state that **“A conservative estimate of 30% prevalence of vaping was adopted based on published South Asian studies [8]”.** We have cited the relevant literature accordingly. Please see below-

**Ref:8:** Pettigrew S, Santos JA, Miller M, Raj TS, Jun M, Morelli G, et al. E-cigarettes: A continuing public health challenge in India despite comprehensive bans. *Prev Med Rep*. 2023; 31:102108. doi:10.1016/j. pmedr.2022.102108
